# Experimental Study on the Influence of Tool Electrode Material on Electrochemical Micromachining of 304 Stainless Steel

**DOI:** 10.3390/ma14092311

**Published:** 2021-04-29

**Authors:** Jianxiao Bian, Baoji Ma, Haihong Ai, Lijun Qi

**Affiliations:** 1 School of Mechanical Engineering, Xi’an Technological University, Xi’an 710021, China; haihongai@126.com (H.A.); qilijun@xatu.edu.cn (L.Q.); 2 School of Mechanical Engineering, Longdong University, Qingyang 745000, China; 3 Shaanxi Key Laboratory of Non-Traditional Machining, Xi’an 710021, China

**Keywords:** electrochemical machining, cathode material, machining quality, oxide film, stray current corrosion

## Abstract

Different cathode materials have different surface chemical components and machining capacities, which may finally result in different machining quality and machining efficiency of workpieces. In this paper, in order to investigate the influence of cathode materials on the electrochemical machining of thin-walled workpiece made of 304 stainless steel, five cylindrical electrodes are used as the target working cathodes of electrochemical machining to conduct experiments and research, including 45# steel, 304 stainless steel, aluminum alloy 6061, brass H62, and tungsten steel YK15. The stray current corrosion, taper, and material removal rate were used as the criteria to evaluate the drilling quality of efficiency of a thin-walled workpiece made of 304 stainless steel. The research results show that from the perspectives of stray current corrosion and taper, aluminum alloy 6061 is an optimal tool cathode, which should be used in the electrochemical machining of thin-walled workpieces made of 304 stainless steel; on the aspect of material removal rate, the 45# steel, 304 stainless steel, and aluminum alloy 6061 present close material removal rates, all of which are higher than that of brass H62 and tungsten steel YK15. Based on comprehensive consideration of both machining quality and machining efficiency, the aluminum alloy 6061 is the best option as the cathode tool in the electrochemical machining of thin-walled workpieces made of 304 stainless steel.

## 1. Introduction

Electrochemical machining technology is a special machining technology different from the traditional machining method. Based on the principle of redox reaction, electrochemical machining can realize the dissolution and removal of metal material on anode workpiece in the ionic state and non-contact form under the external electric field. Because electrochemical machining is not affected by the hardness of metal material, and there is no loss of the cathode tool during machining, it has been widely used in parts manufacturing in the fields of aerospace and molds as well as instruments and apparatuses [1,2]. With the development of the micro electro mechanical system (MEMS), higher requirements are demanded for micro-machining, especially the micro-drilling of thin-walled workpiece. Micro-drilling applying ECM (electrochemical machining) generally has the problem of stray current corrosion. At present, because electrochemical machining is under the impact of stray current corrosion, its machining quality is still a great concern in this field, which needs to be addressed urgently.

In order to improve the quality of electrochemical machining, researchers in China and abroad have made trials on various aspects, including power source, flow field optimization, electrolyte, cathode structure, and machining parameters. According to the characteristics of micro-ECM, Spieser et al. [3] studied the rectangular pulse power supply used in micro-ECM and successfully applied it in experiments. Giandomenico et al. [4] investigated the nanosecond pulse power supply used in micro-ECM and utilized open source hardware to make the pulse generator with adjustable parameters, high precision, and low cost and verified its performance through experiments. To increase the quality of electrochemical machining, many researchers tried to optimize the flow field distribution via multi-physics field simulation, so as to further reduce the influence of non-uniform flow field on electrochemical machining [5,6,7,8,9,10]. Zhan et al. [11] proposed a novel electrochemical machining technique, namely the plasma assisted electrochemical machining technique, and this technique tries to form a thin gas film and plasma layer around the tool electrode by optimizing the potential. Their results show that under certain potential, the gas film formed by PA-ECM can provide side insulation, which can effectively improve the hole machining quality and material removal rate. Hung et al. [12] employed the aluminizing and micro-arc oxidation technique to generate an insulating layer on stainless steel and used it as the tool electrode to conduct an electrochemical machining test. According to their results, side insulation can help improve the machining precision of the hole structure and reduce the stray current corrosion. Furthermore, some scholars tried to improve the electrochemical machining quality by generating an insulating layer on the side of cathode tool, which can significantly alleviate the stray current corrosion of the hole structure. During the electrochemical machining process, the insoluble products in the machining area severely affect the precision of electrochemical machining. In order to improve the update of electrolyte in machining area, researchers have employed techniques such as low-frequency vibration and supersonic vibration to improve the quality of electrochemical machining [13,14,15,16,17,18]. During electrochemical machining, the electrolyte is mainly sodium nitrate or sodium chloride. However, research has shown that a nonlinear electrolyte such as sodium nitrate can provide significantly better machining quality than linear electrolyte-like sodium chloride, and this is mainly due to the passivation effect of sodium nitrate [19]. In order to improve the machining quality of a specific machining object, more scholars choose to optimize machining parameters, such as the machining voltage, duty cycle, impulse frequency, and electrolyte concentration, to achieve better machining quality [20,21,22,23,24,25,26]. At present, research on cathode materials mainly focus on the application of single cathode material in electrochemical machining, such as brass, copper alloy, or iron alloy, while there has been less research in the field of electrochemical machining [27,28,29,30]. Another main reason is that as 304 stainless steel, brass, and tungsten steel are the most common tool electrodes [31], the focus is generally put on the optimization of the cathode structure during the design of the electrochemical machining cathode, while the influence of the cathode material on electrochemical machining is often ignored. Furthermore, different cathode materials also have different chemical components and reaction mechanisms, which result in different machining capacities. Therefore, it is very necessary to study the influence of the cathode material on the electrochemical machining quality.

In order to investigate the influence of the cathode material on the electrochemical machining quality, first, COMSOL Multiphysics 5.3a multi-physics field simulation software was used to simulate the electric field distribution of machining gaps. Then, the five cylindrical electrodes of 45# steel, 304 stainless steel, aluminum alloy 6061, brass H62, and tungsten steel YK15 (among which there are very few applications of aluminum alloy) were used to conduct an experimental study. Next, by using the stray current corrosion, taper, and material removal rate as the criteria to evaluate the drilling quality of thin-walled workpiece made of 304 stainless steel, an SEM (scanning electron microscope) and profile measuring instrument were used to measure the hole.

## 2. Experimental Equipment

**Figure 1:**
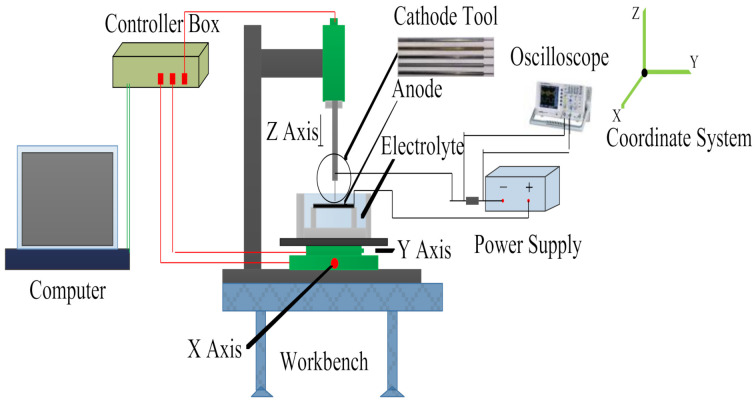
Schematic diagram of electrochemical machining.

The ECM experimental system mainly consists of the machine tool, power system, electrolyte system, motion control, and monitoring system. The air flotation test platform installed in the machine tool can provide certain shock-proof functions, which can effectively ensure stability during the machining process. The machine tool is equipped with a motion control platform, which can realize motion in the three directions of X, Y, and Z, and its positioning precision can reach 0.1 μm. Furthermore, it also includes a motorized spindle which can drive and fixate the cathode tool to make high-speed revolutions. The AN12010D-M single-pulse power supply (Anxi, Wuxi, China) was used as the power supply system. Its voltage can be set within the range of 0–120 V, its frequency is within 10 KHz, and its duty cycle is 0–100%. Compared to the traditional DC power supply, it has the characteristics of intermittent discharge, so it has high machining precision. The motion control and monitoring system was composed of the MP-C154 motion control card, A622 current probe (Tektronix, Shanghai, China), TBS1104 digital storage oscilloscope (Tektronix), and B013 Supereyes (SuperEye, Shenzhen, China). The current probe was mainly used to monitor the current signal in real time, and data were output from the oscilloscope. The key performance indexes of the digital storage oscilloscope include the 100 MHz bandwidth, four channels, and a sampling rate of 1 GS/s. Because the environmental scanning electron microscope (ESEM) can be used to analyze various sample pieces in high vacuum, low vacuum, and environmental vacuum conditions, the ESEM was used to observe our test results. A white light interferometer (WLI) was used to observe the profile of results. The schematic diagram of electrochemical machining is illustrated in Figure 1.

## 3. Simulation of Electric Field

### 3.1. Physical Simulation Model

**Figure 2:**
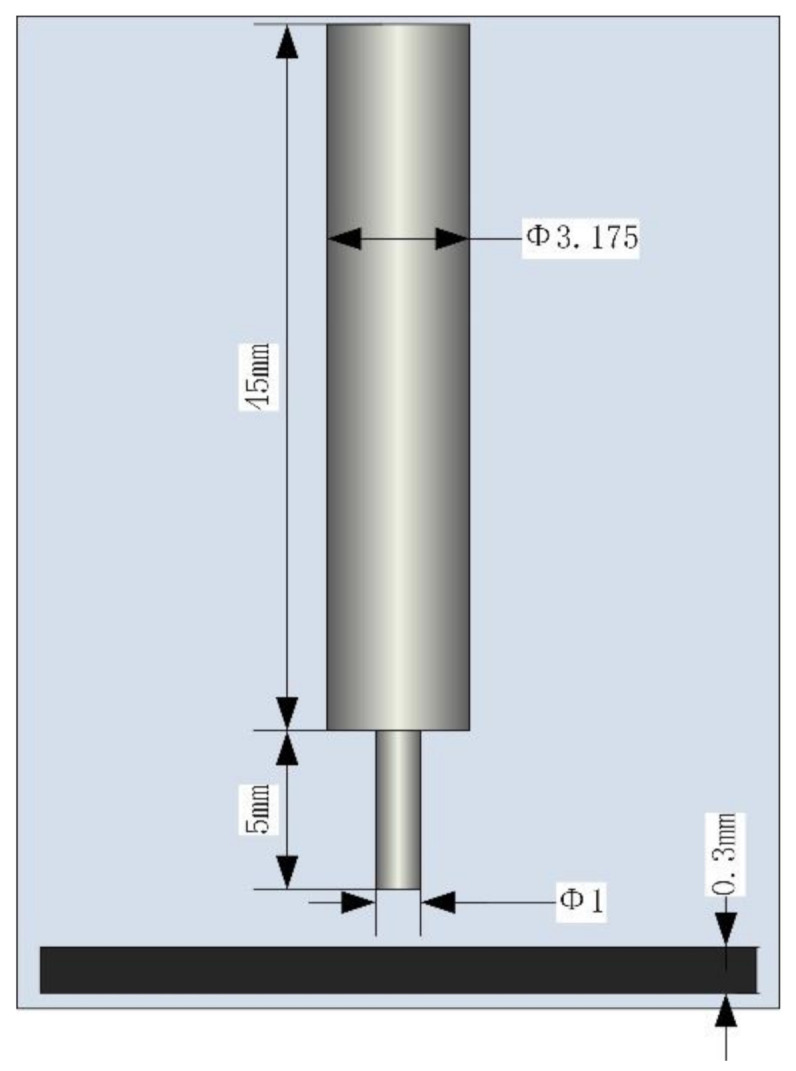
Finite element model.

A finite element model of electrochemical machining was built to study the influence mechanism of different cathode materials on the ECM precision; see Figure 2 for the size and positional relationship of model. For convenience of clamping, the cathode was designed with a stepped construction. The interelectrode gap (IEG) was 0.2 mm.

### 3.2. Physical Simulation Model

For the convenience of building the electric field model, the following assumptions were made for the electric field model during the electrochemical machining process:

(a) The distribution of surface current density on the anode workpiece is decided by the Ohm effect.
(b) The electric conductivity k of the electrolyte is fixed.
(c) The tool electrode is defined as the equipotential surface.

(1) $$\nabla^{2}\varphi =\frac{\partial^{2}\varphi }{\partial^{2}x}+\frac{\partial^{2}\varphi }{\partial^{2}y}+\frac{\partial^{2}\varphi }{\partial^{2}z}=0$$

where φ represents the potential of any point in the electrolyte. The electrode surface can be regarded as an equipotential surface. Therefore, the constant potentials *U_a_* and 0 V are set as the anode boundary $\left(\varphi_{a}=U_{a}=10\;V\right)$ and the cathode boundary $\left(\varphi_{c}=0\;V\right)$, respectively. In addition, all other surfaces are virtual boundaries, which can be regarded as electrically insulating interfaces without current density passing $\left(n\cdot J=\partial \varphi /\partial n=0\right)$.

According to Ohm’s law, the current density J and potential φ have the following relation:

(2) $$J=k\frac{\partial \phi }{\phi n}$$

where n is the unit vector vertical to the workpiece surface, and k is the conductivity of the electrolyte.

In electrochemical machining, the workpiece forming change is decided by the material removal rate on the workpiece surface. According to Faraday’s law, the removal rate of the anode workpiece is

(3) V=ηωJ

where η is the current efficiency, ω is the electrochemical equivalent, and J is the current density.

### 3.3. Simulation and Experimental Parameters

This paper aims to study the influence of cathode material on electrochemical machining. Therefore, in order to prevent other influencing factors from interfering with electrochemical machining, a preliminary basic experiment was conducted on the machinability of an anode workpiece. The results of the basic experiment showed that when the electrolyte temperature was 20 °C, the electrolyte was a mixture of 1 mol/L NaNO_3_ and 0.1 mol/L C_6_H_8_O_7_, the machining voltage was 10 V, the spindle speed was 3000 rpm, the initial interelectrode gap was 0.2 mm, and the feed speed was no higher than 1 μm/s (1 μm/s of feed speed was used to ensure machining efficiency), various cathode materials could remove material from the anode workpiece. Before the electric field simulation of the rotating electrode, it was necessary to set the boundary conditions of the physical field. The parameters of which the initial values needed to be set included potential, cathode speed, and electrolyte conductivity. The electrolyte concentration was determined in the basic experiment, so the electrolyte conductivity could be measured using a conductometer. Table 1 lists the parameters and values of the simulation model and experiments. The conductivity of electrolytes was measured using a DDS-307 conductivity meter (Shanghai INESA Scientific Instrument Co., Ltd., Shanghai, China). The boundary conditions of the electric field were as follows: the 10 V potential was connected to the anode workpiece, and the cathode was connected to ground. The boundary conditions of the flow field were as follows: the electrolyte flow model chosen was turbulent flow, and there was no inflow or outflow. (In this paper, because the electrochemical machining was conducted in the hydrostatic state, and the spindle speed was 3000 rpm, high-speed rotation caused the flow of electrolyte, so it was turbulent flow). The steady-state solver with strong applicability in COMSOL Multiphysics was selected.

### 3.4. Simulation Results

**Figure 3:**
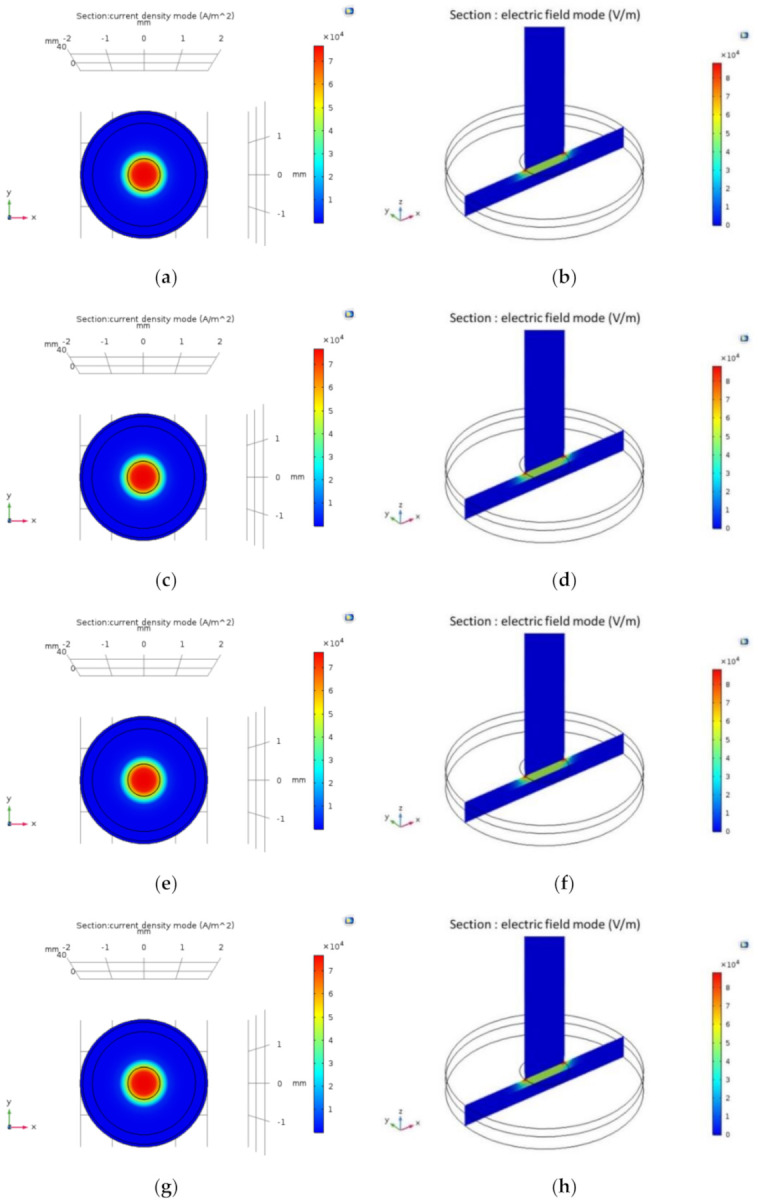

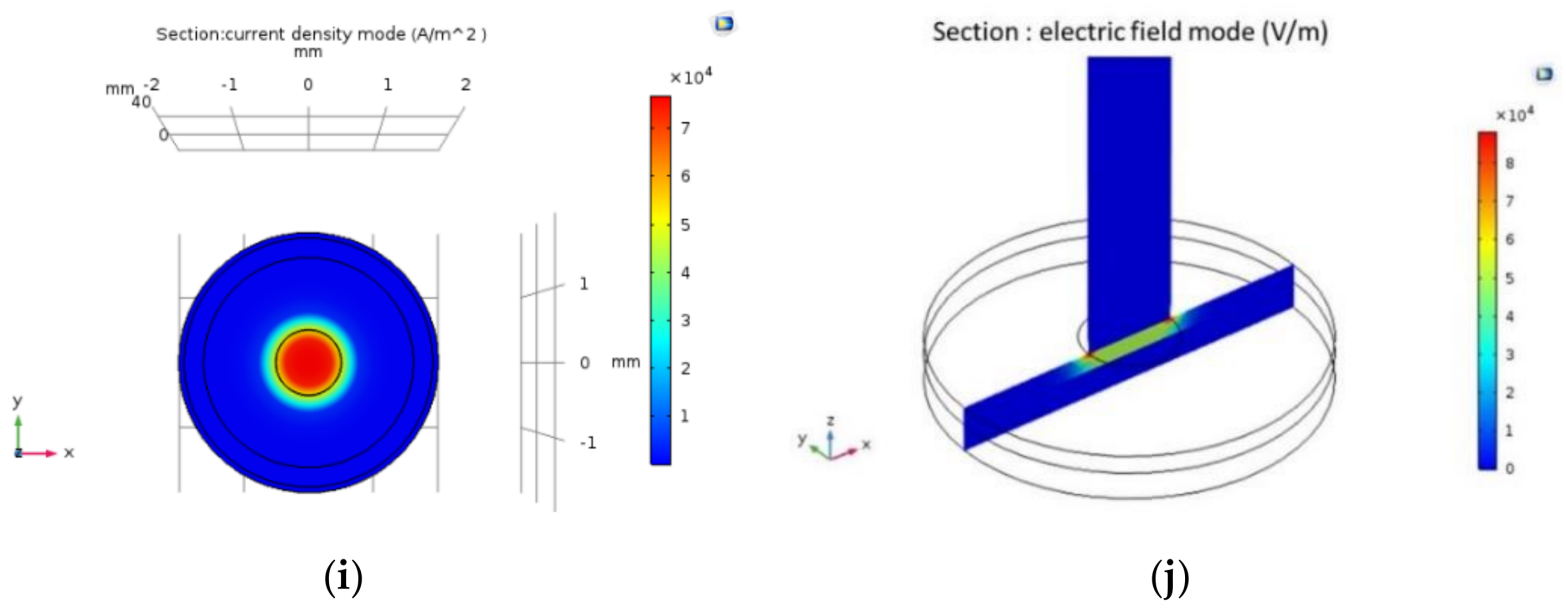
Current density distribution on anode surface with different cathode materials: (**a**) 45# steel; (**c**) 304 stainless steel; (**e**) aluminum alloy 6061; (**g**) brass H62; (**i**) tungsten steel YK15. Electric field distribution on anode surface with different cathode materials: (**b**) 45# steel; (**d**) 304 stainless steel; (**f**) aluminum alloy 6061; (**h**) brass H62; (**j**) tungsten steel YK15.

In order to study the current density and electric field distribution of different cathode materials on the anode surface and the differences among these materials, the anode was 304 stainless steel in the simulation model. The electrolyte was the mixed solution of NaNO_3_ and C_6_H_8_O_7_, and the current density mode and electric field mode on the interface between anode and electrolyte were used as the simulation results. Figure 3 shows the distributions of current density mode and electric field mode on the anode surface when the cathode materials were 45# steel, 304 stainless steel, aluminum alloy 6061, brass H62, and tungsten steel YK15, respectively. According to the simulation results, the current density mode and electric field mode generated by various cathode materials on the anode surface were close.

## 4. Experimental Results and Discussion

In order to study the influence of cathode material on the electrochemical machining of the hole, five electrodes of the same size and shape were used, and they were made of 45# steel, 304 stainless steel, aluminum alloy 6061, brass H62, and tungsten steel YK15, respectively. Three experiments were conducted using each cathode tool to prevent accidental experimental error. The stray current corrosion refers to the cellular structure generated around the hole, which would severely affect the surface quality of the hole. Taper is a very important criterion used to evaluate the machining quality of a straight hole, and the smaller the taper is, the better the hole machining quality. For a through-hole, the machining gap refers to the side gap of the hole, and this gap is half of the difference between the hole diameter and the cathode tool diameter. The computational formula of hole taper is

(4) $$\tan\alpha =\frac{d_{1}-d_{0}}{2h}$$

where *d*_1_ is the outlet diameter of the hole; *d*_0_ is the inlet diameter of the hole; and h is the thickness of the stainless steel plate.

The computational formula of the removal rate is

(5) $$MRR=\frac{V_{0}-V_{1}}{V_{0}}$$

where *V*_0_ is the workpiece size before machining; and *V*_1_ is the workpiece size after machining.

Because it is difficult to calculate the workpiece size after machining, for the convenience of calculation, size can therefore be converted to mass for the calculation of material removal rate. The computational formula is as follows:

(6) $$MRR=\frac{\left(V_{0}-V_{1}\right)\rho }{V_{0}\rho }=\frac{M_{0}-M_{1}}{M_{0}}$$

where ρ is the density of metal material; *M*_0_ is the workpiece mass before machining; and *M*_1_ is the workpiece mass after machining.

### 4.1. The Stray Current Corrosion of Hole

**Figure 4:**
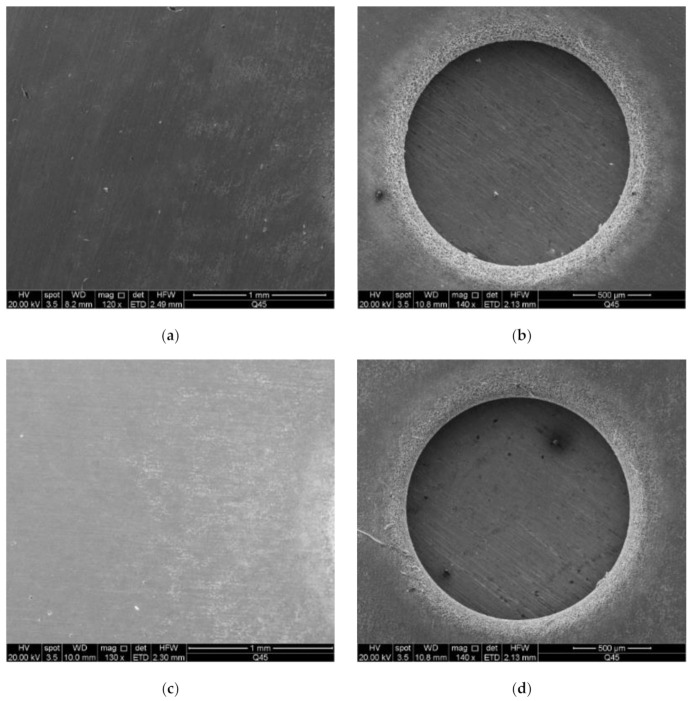

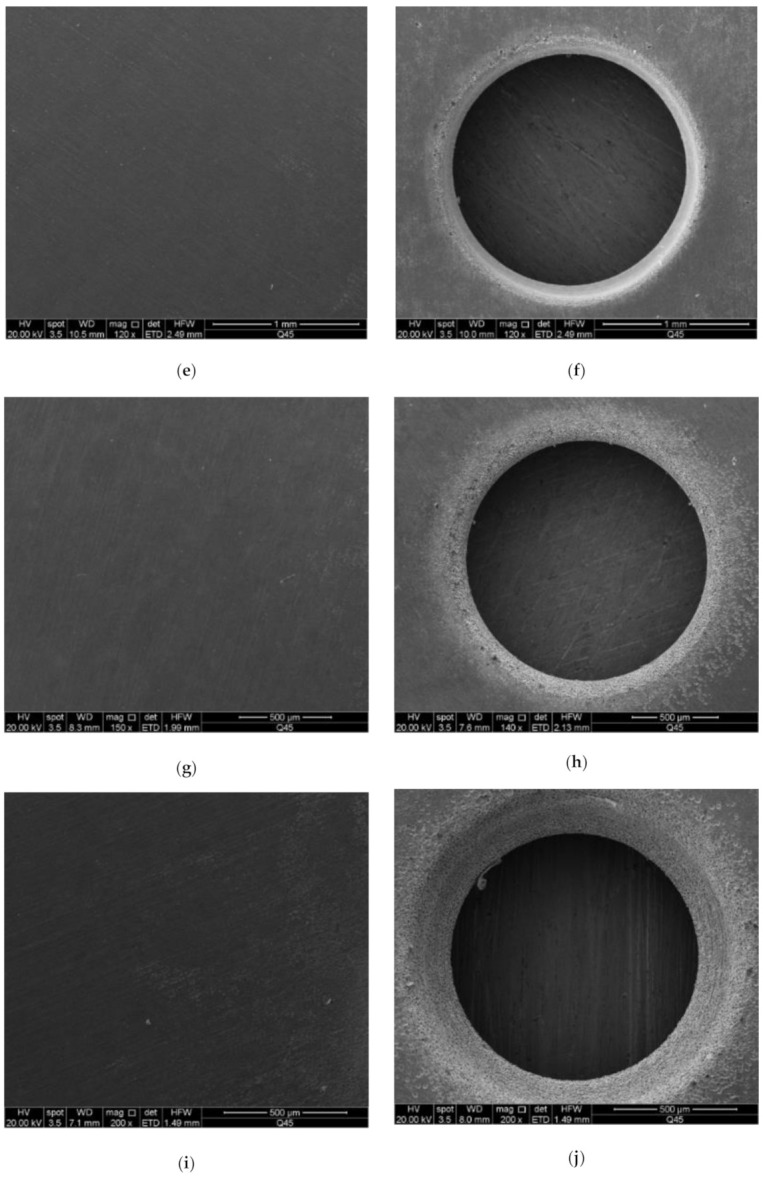
SEM images of the surface before machining with the 304 stainless steel anode when different cathode material was used: (**a**) 45# steel; (**c**) 304 stainless steel; (**e**) aluminum alloy 6061; (**g**) brass H62; (**i**) tungsten steel YK15. SEM images of upper hole with the 304 stainless steel anode when different cathode material was used: (**b**) 45# steel; (**d**) 304 stainless steel; (**f**) aluminum alloy 6061; (**h**) brass H62; (**j**) tungsten steel YK15.

Figure 4 shows the SEM images of 304 stainless steel when different cathode material was used before and after machining. According to Figure 4, the quality of the hole obtained through machining using aluminum alloy was significantly better than that obtained using the 45# steel, 304 stainless steel, brass H62, or tungsten steel YK15, and the surrounding stray current corrosion was also lighter when the aluminum alloy was used as the cathode material. This is because it is not easy for 45# steel, 304 stainless steel, brass H62, or tungsten steel YK15 to oxidize in the ambient environment, which means a dense oxide film generally will not be formed on their surface under room temperature. On the contrary, when the aluminum electrode is exposed to air, a dense oxide film tends to form on its surface. Therefore, during electrochemical machining, the oxide film generated on the side of the aluminum electrode can provide sidewall insulation, and additional stray current, which may cause secondary corrosion to the metal around the hole, will not be generated.

### 4.2. Taper of Hole

**Figure 5:**
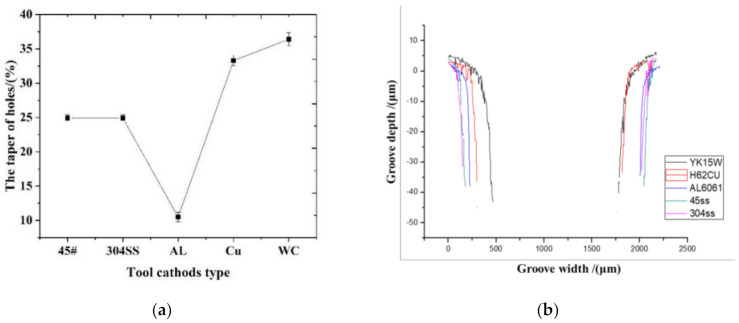
(**a**) The influence of tool cathode type on hole taper; (**b**) 2D profiles of corresponding holes of different cathode materials.

Figure 5 shows the influence of tool cathode type on hole taper. The inner wall outline and size of hole were obtained using a white light interferometer. According to Figure 5a, the tapers of holes were in the following order of cathodes: aluminum alloy 6061 < 45# steel = 304 stainless steel < brass H62 < tungsten steel YK15. The tapers were close when 45# steel and 304 stainless steel were used as the cathodes. However, the taper of the hole machined using the aluminum alloy 6061 was slow to change, the holes near the outlet were basically straight holes, and the machining results were great. The reasons may be that (a) during the machining process, the aluminum alloy 6061 bonded with oxygen in water and generated a dense oxide film on its surface, which prevented further corrosion of the anode surface by the cathode tool; and (b) furthermore, the aluminum alloy 6061 had a passivation reaction in the passivating electrolyte of sodium nitrate solution. Therefore, in the future, aluminum alloy 6061 can be used as the tool electrode in the electrochemical machining of 304 stainless steel.

### 4.3. Material Removal Rate

**Figure 6:**
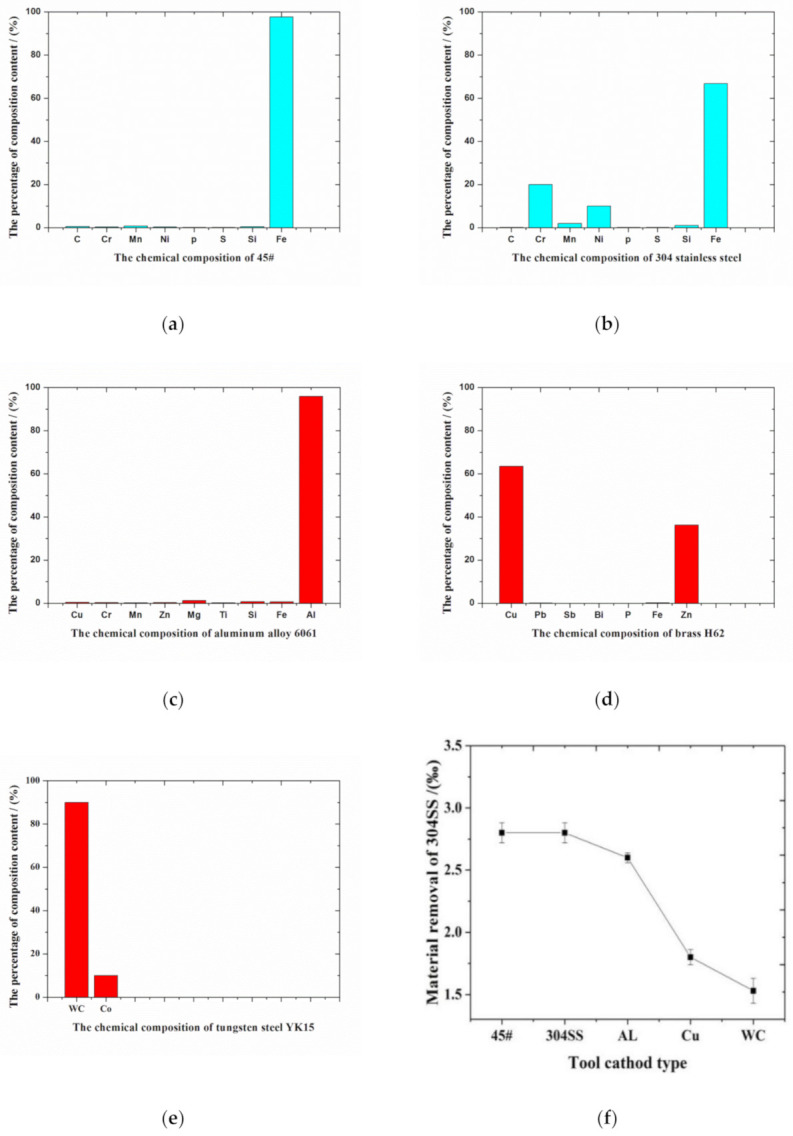
Chemical components of different cathode materials and their contents: (**a**) 45# steel; (**b**) 304 stainless steel; (**c**) aluminum alloy 6061; (**d**) brass H62; (**e**) tungsten steel YK15; (**f**) material removal rate of corresponding anode workpiece made of 304 stainless steel with different cathode materials.

According to the profiles shown in Figure 5b and Figure 6f, the machining gap and material removal rate were in the following order of cathode materials: 45# steel = 304 stainless steel > aluminum alloy 6061 > brass H62 > tungsten steel YK15. It is well known that a bigger machining gap represents stronger machining capacity of the tool electrode. Therefore, 45# steel and 304 stainless steel had the strongest machining capacity of the anode workpiece, which also had the highest material removal rate, while tungsten steel had the weakest machining capacity of the anode workpiece. The material removal rates of 45# steel and 304 stainless steel were slightly higher than that of aluminum alloy 6061. Figure 6 shows that compared to other cathode materials, the tungsten steel YK15 mainly consists of compound WC and metal Co., and the content of WC is approximately 90%, while the metal contents of the rest of cathode materials are nearly 100%. Therefore, the tungsten steel YK15 has weaker machining capacity than the other cathode materials. The brass H62 tends to have severe corrosion in corrosive media under high humidity, that is, the passive film of brass tends to be damaged in H^+^ and NO_3_^−^ solutions. In solution, brass tends to be oxidized into cuprous oxide, which will be combined with a small amount of oxygen in the solution and generate copper oxide. However, the oxide film will be dissolved in the acid environment. With the corrosion of brass H62, the gap between the brass H62 and anode workpiece made of 304 stainless steel will increase, which will gradually weaken its machining capacity. In comparison, the aluminum alloy 6061 presents great resistance to corrosion, and the material surface is dense without defects after machining, presenting a great oxidation effect. Therefore, based on comprehensive consideration of machining quality and material removal rate, the aluminum alloy 6061 is definitely a better choice as the cathode used in electrochemical machining of 304 stainless steel.

## 5. Conclusions

In order to investigate the influence of electrode material on the electrochemical machining of thin-walled workpiece made of 304 stainless steel, we studies the electric field distribution of the machining gap and used the five tool cathodes of 45# steel, 304 stainless steel, aluminum alloy 6061, brass H62, and tungsten steel YK15 (among which there are very few applications of aluminum alloy) to conduct experiments. According to the simulation and experimental results, we can reach the following conclusions:

1. Simulation was conducted to the current density and electric field distribution on the anode surface, and the results show that the variation of the cathode tool could not change the current density mode and electric field mode generated on the anode surface.
2. According to the simulation and experimental results, the oxide film generated on the aluminum electrode surface could provide side insulation. In the machining of the 304 stainless steel workpiece, the tool cathode of aluminum alloy 6061 could provide better machining quality than other common tool electrodes. In addition, the oxide film generated on the aluminum electrode surface could alleviate secondary corrosion of the anode workpiece, which could help reduce the taper of the hole structure, and the stray current corrosion could also be reduced. Combining the aspects of machining quality and material removal rate, aluminum alloy 6061 is a better choice of cathode used in the electrochemical machining of 304 stainless steel.
3. The insulation characteristics of its oxide film can facilitate the promotion and application of the aluminum alloy 6061 tool cathode in the field of electrochemical machining.

## Figures and Tables

**Table 1:** Parameters and values of simulation model and experiments.

Parameters	Values
Cathode material	H62 brass|6061 aluminum|304 stainless steel 45 steel|YK15 tungsten steel
Anode material	304 stainless steel
Cathode size (mm)	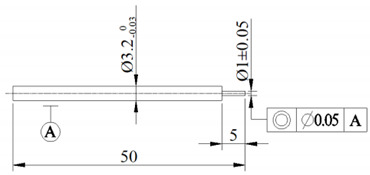
Anode size (mm)	25 × 25 × 0.3
Electrolyte	1 mol/L NaNO_3_ + 0.1 mol/L C_6_H_8_O_7_
Cathode potential (V)	10
Anode potential (V)	0
Initial interelectrode gap (IEG) (mm)	0.2
Electrolyte conductivity (S/m)	1.542
Temperature (°C)	20
Spindle speed (rpm)	3000
Feed speed (μm/s)	1.0
Workpiece thickness(mm)	0.3

## Data Availability

Data sharing is not applicable to this article.

## References

[1] Razali A.R., Qin Y. (2013). A Review on Micro-manufacturing, Micro-forming and Their Key Issues. Procedia Eng..

[2] Bian J.X., Ma B.J., Liu X.F. (2020). Experimental Study of Tool Wear in Electrochemical Discharge Machining. Appl. Sci..

[3] Spieser A., Ivanov A. (2015). Design of a pulse power supply unit for micro-ECM. Int. J. Adv. Manuf. Technol..

[4] Giandomenico N., Meylan O. (2016). Development of a New Generator for Electrochemical Micro-machining. Procedia CIRP.

[5] Xu Z.Y., Sun L.Y., Hu Y. (2014). Flow Field Design and Experimental Investigation of Electrochemical Machining on Blisk Cascade Passage. Int. J. Adv. Manuf. Technol..

[6] Qu N.S., Hu Y., Zhu D. (2014). Electrochemical Machining of Blisk Channels with Progressive-Pressure Electrolyte Flow. Mater. Manuf. Process..

[7] Dabrowski L., Paczkowski T. (2005). Computer Simulation of Two-Dimensional Electrolyte Flow in Electrochemical Machining. J. Electrochem..

[8] Kozak J., Dabrowski L., Lubkowski K. (2000). CAE-ECM System for Electrochemical Technology of Parts and Tools. J. Mater. Process. Technol..

[9] Fujisawa T., Inaba K., Yamamoto M. (2008). Multiphysics Simulation of Electrochemical Machining Process for Three-Dimensional Compressor Blade. J. Fluid. Eng..

[10] Zhang C., Ai H., Yan Z., Jiang X., Cheng P., Hu Y., Tian H. (2020). Cathode optimization and multi-physics simulation of pulse electrochemical machining for small inner-walled ring grooves. Int. J. Adv. Manuf. Technol..

[11] Zhan S.D., Zhao Y.H. (2020). Plasma-assisted electrochemical machining of microtools and microstructures. Int. J. Mach Tool. Manuf..

[12] Hung J.C., Liu Y.R., Tsui H.P. (2019). Electrode insulation layer for electrochemical machining fabricated through hot-dip aluminizing and microarc oxidation on a stainless-steel substrate. Surf. Coat. Technol..

[13] Wang F., Yao J., Kang M. (2020). Electrochemical machining of a rhombus hole with synchronization of pulse current and low-frequency oscillations. J. Manuf. Process..

[14] Lian H., Guo Z., Huang Z. (2013). Experimental Research of Al6061 on Ultrasonic Vibration Assisted Micro-Milling. Procedia CIRP.

[15] Natsu W., Nakayama H., Yu Z. (2012). Improvement of ECM characteristics by applying ultrasonic vibration. Int. J. Precis. Eng. Manuf..

[16] Yang I., Min S.P., Chu C.N. (2009). Micro ECM with ultrasonic vibrations using a semi-cylindrical tool. Int. J. Precis. Eng. Manuf..

[17] Pei W., Yu Z., Li J. (2013). Influence of Abrasive Particle Movement in Micro USM. Procedia CIRP.

[18] Yao Z., Guo Z.N., Zhang Y.J. (2013). Research on the Frequency Tracking in Rotary Ultrasonic Machining. Procedia CIRP.

[19] Shibuya N., Ito Y., Natsu W. (2012). Electrochemical machining of tungsten carbide alloy micro-pin with NaNO3 solution. Int. J. Precis. Eng. Manuf..

[20] Cole K.M., Kirk D.W., Singh C.V. (2017). Optimizing electrochemical micromachining parameters for Zr-based bulk metallic glass. J. Manuf. Process..

[21] Rathod V., Doloi B., Bhattacharyya B. (2015). Experimental Investigations into Machining Accuracy and Surface Roughness of Microgrooves Fabricated by Electrochemical Micromachining. Proc. Inst. Mech. Eng. Part B J. Eng. Manuf..

[22] Abuzied H.H., Awad M.A., Senbel H.A. (2012). Prediction of Electrochemical Machining Process Parameters Using Artificial Neural Networks. Int. J. Comput. Sci. Eng..

[23] ChenthilJegan T.M., Ravindran D. (2017). Electrochemical Machining Process Parameter Optimization Using Particle Swarm Optimization. Comput. Intell..

[24] Senthilkumar C., Ganesan G., Karthikeyan R. (2011). Parametric Optimization of Electrochemical Machining of Al/15%SiCp Composites Using NSGA-II. Trans. Nonferrous Met. Soc. China.

[25] Chakradhar D., Gopal A.V. (2011). Multi-objective Optimization of Electrochemical Machining of EN31 Steel by Grey Relational Analysis. Int. J. Model. Optim..

[26] Mukherjee R., Chakraborty S. (2013). Selection of the Optimal Electrochemical Machining Process Parameters Using Biogeography-based Optimization Algorithm. Int. J. Adv. Manuf. Technol..

[27] Thangamani G., Kalaichelvan K., Thangaraj M. (2016). Influence of Coated Tool Electrode on Drilling Inconel Alloy 718 in Electrochemical Micro Machining. Procedia CIRP.

[28] Meng L.C., Zeng Y.B., Zhu D. (2018). Helical Carbon Nanotube Fiber Tool Cathode for Wire Electrochemical Micromachining. J. Electrochem. Soc..

[29] Niu S., Qu N.S., Fu S.X., Fang X.L., Li H.S. (2017). Investigation of inner-jet electrochemical milling of nickel-based alloy GH4169/Inconel 718. Int. J. Adv. Manuf. Technol..

[30] Liu J., Wang H., Zhu D. (2018). Electrochemical Machining of γ-TiAl Intermetallic Blades by Using the Stainless Steel Anti-copied Tool Electrodes. Procedia CIRP.

[31] Rahman Z., Das A.K., Chattopadhyaya S. (2018). Microhole drilling through electrochemical processes: A review. Adv. Manuf. Process..

